# Liposome-Fe_3_ O_4_-Doxorubicin Mediated Treatment of Melanoma Tumors

**DOI:** 10.34172/apb.2023.034

**Published:** 2022-01-02

**Authors:** Azalia Azlegini, Sirus Javadpour, Mohamad Ebrahim Bahrololoom

**Affiliations:** ^1^Laboratory of Materials Science & Engineering, School of engineering, Zand Blvd. 7134851154, Shiraz University, Iran.; ^2^Department of Materials Science & Engineering, School of engineering, Zand Blvd. 7134851154, Shiraz University, Iran.

**Keywords:** Doxorubicin, Hyperthermia, Liposome, Magnetic nanoparticles, Melanoma tumor

## Abstract

***Purpose:*** Magnetic hyperthermia is a treatment method based on eddy currents, hysteresis, and relaxation mechanisms of magnetic nanoparticles (MNPs). MNPs such as Fe_3_ O_4_ have the ability to generate heat under an alternating magnetic field. Heat sensitive liposomes (Lip) convert from lipid layer to liquid layer through heat generated by MNPs and can release drugs.

***Methods:*** In this study, different groups of doxorubicin (DOX), MNPs and liposomes were evaluated. The MNPs were synthesized by co-precipitation method. The MNPs, DOX and a combination of MNPs and DOX were efficiently loaded into the liposomes using the evaporator rotary technique. Magnetic properties, microstructure, specific absorption rate (SAR), zeta potential, loading percentage of the MNPs and DOX concentration in liposomes, in vitro drug release of liposomes were studied. Finally, the necrosis percentage of cancer cells in C57BL/6J mice bearing melanoma tumors was assessed for all groups.

***Results:*** The loading percentages of MNPs and concentration of DOX in the liposomes were 18.52 and 65% respectively. The Lip-DOX-MNPs at the buffer citrate solution, showed highly SAR as the solution temperature reached 42°C in 5 minutes. The release of DOX occurred in a pH-dependent manner. The volume of tumor in the therapeutic groups containing the MNPs significantly decreased compared to the others. Numerical analysis showed that the tumor volume in mice receiving Lip-MNPs-DOX was 9.29% that of the control and a histological examination of the tumor section showed 70% necrosis.

***Conclusion:*** The Lip-DOX-MNPs could be effective agents which reduce malignant skin tumors growth and increase cancer cell necrosis.

## Introduction

 Hyperthermia has been introduced as a cancer complementary therapy to reduce the side effects of the commonly used chemotherapy.^1-5^ In magnetic hyperthermia, the magnetic nanoparticles (MNPs) are exposed to a magnetic field to provide the required energy for temperature rise.^6-10^ The temperature (around 42-44℃) damages the tumor cells without any significant injury to the tissues. Depending on the crystalline degree and the size of the nanoparticles, the therapeutic performance be change.^10,11^ Till now, many kinds of magnetic structures have been introduced for hyperthermia purposes including magnetite, spinel ferrites, and manganese-based perovskite structures.^12-15^ The Fe_3_O_4_ nanoparticles have been the best option for biomedical applications because of high thermal resistance, chemical stability, and high saturation magnetization.^11,16,17^ The Fe_3_O_4_-based heat can be generated through three mechanisms of eddy current, hysteresis loss, and relaxation loss. The latter mechanism is the main contributor of the heat loss required in hyperthermia.^18,19^ The Neel or Brown relaxation mechanisms are responsible for heat generation by MNPs.^20^ In the Neel mechanism, the super spin rotates and orients to the direction parallel to the applied field but in the Brown mechanism the super spin remains fixed relative to the crystal orientation. To improve treatment efficacy, the MNPs were modified for simultaneous targeted drug delivery and magnetic hyperthermia. This strategy has become very popular in the pharmaceutical industry. The MNPs were modified with liposomes to encapsulate drugs.^21^ Indeed, liposomes have been used as nanocarriers and chemotherapeutic drug carriers.^22,23^ The liposomes are lipid bilayer spherical vesicles capable of loading a large quantity of hydrophobic and hydrophilic drugs in their lipid bilayer or cores.^17,24,25^ The liposomes can be thermo sensitive for controlled drug release through lipid bilayer transition from gel to liquid phase upon heating. The toxic drugs can be carried to the right environment without significant release into the external media.^26,27^ In addition, for drug delivery purposes, biocompatibility and biodegradability properties are essential and liposomes have both factors.^28^ DOX is one of the chemotherapy drugs used to treat cancer. It is also the first approved drug based on the liposome system for the treatment of patients.^29,30^ The first Lip-DOX injection was approved in 1995 by the FDA.^30^ DOX is encapsulated in the aqueous layer of liposomes^31^ and released at the melting phase transition temperature in the range of 40-45°C.^30,32,33^ In the present research, the Lip-MNPs-DOX was synthesized and then used to treat melanoma cancer.^34^ The hyperthermia effect was studied in-vivo to find out tumor size, necrosis percentage, and anti-tumor efficacy. In fact, the MNPs were used for the dual purpose of firstly heat creation to accelerate the leakage of the drug from the liposome and secondly to create magnetic hyperthermia.

## Materials and Methods

###  Chemicals 

 The initial materials consisting of iron (III) chloride hexahydrate (FeCl_3_.6H_2_O) and iron (II) chloride tetrahydrate (FeCl_2_.4H_2_O) were purchased from Merck (Darmstadt, Germany) and used as received without any further purification. Ammonia solution (NH_3_) and Citric acid (C_6_H_8_O_7_) were purchased from Merck as a reduction agent and dispersant agent respectively. Doxorubicin (DOX) hydrochloride ( > 99%) was purchased from the Reza Hospital (Shiraz, Iran). Dipalmitoyl phosphatidylcholine (DPPC), cholesterol (CH), and distearoyl-phosphatidylethanolamine-methyl poly thyleneglyconjugate-2000 (mPEG2000-DSPE) were provided by the Research Center for New Technologies in Life Science Engineering, University of Tehran, Iran.

###  Cells and animals

 Thirty-five female C57BL/6J mice (5-6 weeks, 15-20 g) were purchased from the Pasteur Institute of Iran. All animals were used in this study in accordance with international medical ethics and institutional guidelines. Prior to the melanoma cell injection into the mice, the cell viabilities were assessed by microscopic observation of the cells mixed with trypan blue. The mice were divided into seven groups (5 mice in each group) and the melanoma cells (B16/F10) were injected into the left leg subcutaneously on day 1. The tumor volumes were allowed to grow up to 150 mm^3^ and then the treatment was started according to the procedure presented in Table 1 by intramuscular injection. The tumor size was monitored on day 13, 16, 19, 25, and 28 by caliper. The volume of tumor was calculated by 0.5(width)^2^ in length.^17^ According to Table 1 compounds were injected four times every 3 days and immediately after each injection, the mice were exposed to an alternating magnetic field in the hyperthermia device. In the control group only saline normal was injected four times every 3 days.

**Table 1:** Treatment type and dosage per group

**Group**	**Name**	**Dose of injection**	**Hyperthermia**
Group 1	MNPs	0.1 cc	10 min hyperthermia
Group 2	Lip-MNPs-DOX	0.1 cc	15 min hyperthermia
Group 3	Lip	0.2 cc	Without hyperthermia
Group 4	DOX	0.02 cc	Without hyperthermia
Group 5	Lip-MNPs	0. 2 cc	15 min hyperthermia
Group 6	Lip-DOX	0.1 cc	Without hyperthermia
Group 7	Control	0.2 cc	Without hyperthermia

###  Synthesis of iron oxide MNPs

 Fe_3_O_4_ nanoparticles were synthesized by co-precipitation method. First, FeCl_3_.6H_2_O and FeCl_2_.4H_2_O were weighed according to stoichiometric ratio and were dissolved in deionized water. The NH_3_ was added to the solution for adjusting the pH to 10.5. The nanoparticles were synthesized by the reduction of metallic salts in the presence of ammonia as the reducing agent. The solution was then mixed using a stirrer at 80°C for 1 hour. Next, a specific amount of citric acid (1 mg/ml) was added to the solution and stirred for 1 hour. The synthetic particles were separated by magnet and the solution was thrown away. Then they were washed with deionized water and ethanol six times. The particles were finally left to air dry for 24 hours.^35^

###  Synthesis of Lip-MNPs-DOX, Lip-MNPs and Lip-DOX

 DPPC: mPEG2000-DSPE:cholesterol with a mass ratio of 86:4:10 were dissolved in 3 mL ethanol 99%. The solution was evaporated by rotary evaporator at 38°C to reach the thin film of lipid. The film was dehydrated after 20 minutes.^36,37^ The mixture of 10 mL citrate buffer (0.01 M, pH 6.2) and 20 mg magnetite nanoparticles were dispersed by ultrasonification for 10 minutes and then added to the balloon in the rotary evaporator to obtain the Lip-MNPs.^36,37^ The Lip-MNPs were then obtained by centrifugation for 10 minutes. The non-encapsulated MNPs were separated by an Amicon filter (100 kD). Finally samples were dispersed by ultrasonification. For synthesis of Lip-MNPs-DOX, the mixture of citrate buffer and DOX 100:8 molar ratio were added to the thin film (Lip-MNPs) drop by drop at 40°C and were stirred for 4 hours then kept in the refrigerator for analyses. Unloaded DOX were removed by dialysis. The Lip-DOX was synthesized similar to the Lip-MNPs and Lip-MNPs-DOX preparation method and only MNPs were removed.^31^

###  Characterization

 The crystallographic analysis of Fe_3_O_4_ was done by X-ray diffraction (Bruker D8 Advanced, XRD, Germany and USA) with 2θ angle of 10-70°. The magnetic properties of Fe_3_O_4_ were studied at room temperature using a vibrating sample magnetometer (Meghnatis Daghigh Kavir, VSM, Kashan, Iran). The microstructure of samples was studied using a transmission electron microscope (Zeiss, Em10c -100Kv, TEM, Germany) model at an operating voltage of 100 kV. The average particle size, polydispersity index, and average zeta (z)-potential of Lip, Lip-MNPs-DOX, Lip-DOX, Lip-MNPs, and MNPs were all determined by the dynamic light scattering technique (Malvern Instruments Ltd, DLS, UK). For the DSL measurements, the samples were diluted to a low concentration and results were collected with a light scattering angle of 90° and a holder temperature of 25°C. The topology of the liposomes was investigated by scanning electron microscopy (Leica Cambridge S360, SEM, UK). The loading percentage of the MNPs in liposome was determined by ICP-OES (Varian, model Vistapro ICP-OES, USA). The DOX concentration of the liposome was determined by high performance liquid chromatography (Agilent Technologies, HPLC, Palo Alto, CA). The mobile phase was methanol 58%, potassium phosphate 41% and acetic acid 1%.

###  Specific absorption rate (SAR) tests

 The thermal behavior of MNPs was investigated using a hyperthermia device, by the Laboratory of Material Engineering, Shiraz University. The samples with magnetic nano-particles were subjected to a hyperthermia test before injection. Therefore, solutions of Lip-DOX-MNPs, Lip-MNPs and MNPs (MNPs = 0.1 g, 0.2 g and 0.4 g) at the same concentration of buffer citrate were located into a device with power of 1000 Watt, of frequency 405 kHz. The heat-generating capability of MNPs was determined by measuring the SAR in different groups.^14^ Where C is specific heat of the ferrofluid, ΔT/Δt is the initial slope of the time-dependent temperature curve, and m_ferrite_ is the total ferrite content in the fluid.^14,38^

 SAR = C × (∆ T/∆ t) × (1/m _ferrite_)

###  In vitro drug release

 DOX release was determined in the plasma and tumor environment. Two kinds of buffer phosphate with pH 7.4 and 5.5 were chosen close to the pH of the tumor and blood.^39,40^ For this purpose, 1.0 mL Lip-MNPs-DOX was poured into dialysis bags and incubated in 30 mL of buffer at a temperature 37°C ^40^ and a stirring speed of 100 rpm. After a predefined time period, 2 mL of buffer was removed for analysis and 2 mL of fresh buffer replaced it. The optical absorption of the sample was measured individually with a UV/VIS spectrophotometer and the concentration of drug released into the media was calculated by a standard curve method.

###  In vivo protocol 

 The tumor model was established by subcutaneously implanting B16/F10 cells into the left leg. By the twelfth day, the volume of tumors had increased exponentially and the treatment was started in the different groups. The tumor volume was measured every 3 days until day 28 in all groups. All mice were sacrificed on the 28^th^ day and tumors were excised and fixed in 0.1 M formalin for 48 hours. The samples were then embedded in paraffin and the sections were stained with a hematoxylin and eosin (HE) solution using the protocol of Masson’s trichrome stain, Sigma Accustain Trichrome Stain Kit. The cuts were washed in water and differential solution and put on microscope slides for observation under high power field microscopy at 40X magnification.

## Results and Discussion

###  Characterization of Lip-MNPs-DOX

**Figure 1:**
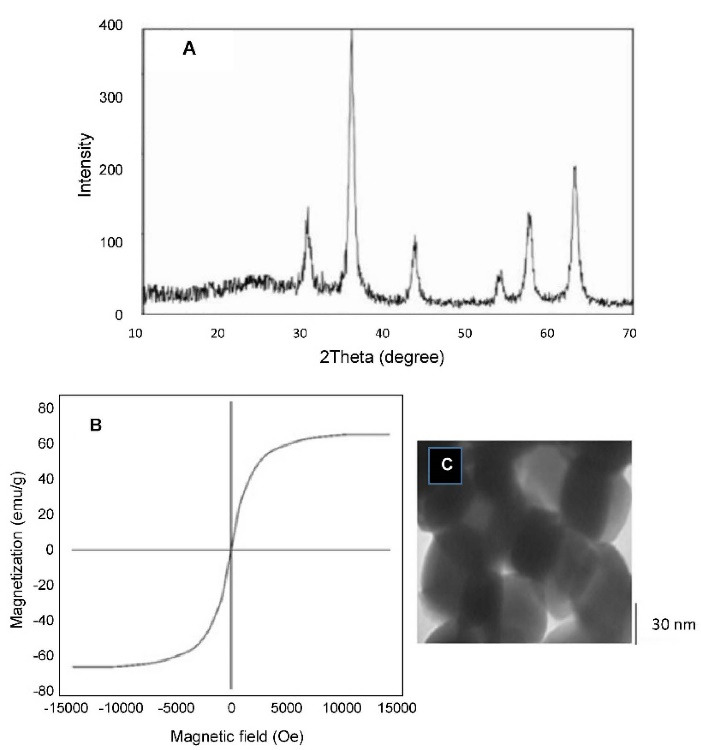
(A) X-ray pattern, (B) M-H curves of Fe_2_O_3_ nanoparticles, (C) TEM image of Fe_3_O_4_ particles.

 The XRD pattern of Fe_3_O_4_ nanoparticles was shown in Figure 1A. The peaks appearing at various 2θ angles are related to the planes of [220], [311], [400], [422], [440], and [511], which represent the inverse spinel crystal planes of magnetite and the intensities and positions of peak in XRD patterns match with standard spinel structure.^41^ The absence of peaks at [110], [200], [211], and [533], which refers to the middle phases, indicates that the target phase was reached without impurities. The crystalline structure of the nano particles confirms that we have synthesized pure Fe_3_O_4_ with a cubic structure.

 The magnetization curves of the Fe_3_O_4_ nanoparticles showed typical features of super paramagnetic behavior (Figure 1B). The saturation magnetization (Ms), specific remnant magnetization (Mr), and coercivity (Hc) measured 63/29 emu g^-1^, 2/99 emu g^-1^, and 2/20 Oe, respectively. We have succeeded in synthesizing magnetic nano-particles with high saturation magnetization and the partial amounts of Mr and Hc confirms the creation of Fe_2_O_3_ super paramagnetic structure.^37,41^

**Figure 2:**
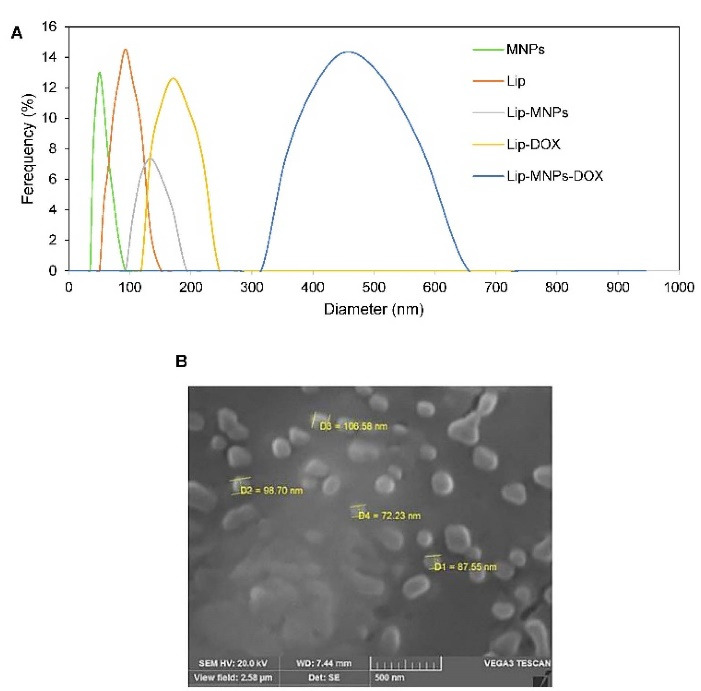
(A) Particle size distribution in different groups: Lip-DOX-MNPs, Lip-DOX, Lip-MNPs, Lip, and MNPs, (B) SEM image of liposomes.

**Figure 3:**
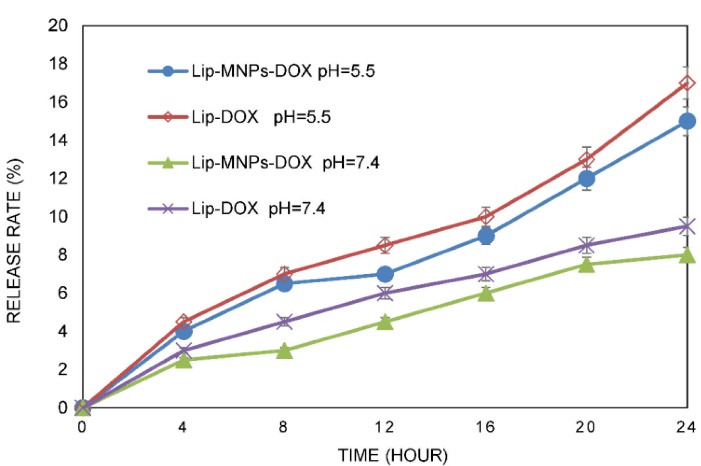
Release profile of DOX from liposome in vitro (The data represent mean ± standard deviation).

 The transmission electron microscope was used for observing the microstructure and size of Fe_3_O_4_ (Figure 1C). According to Figure 1C, the sizes of the particles were measured to about 40 nm. The size distribution of the Lip-MNPs-DOX, MNPs, Lip-DOX, Lip-MNPs, and Lip are all shown in Figure 2A. According to DLS analyses, the average size is presented in Table 2. The size range of liposomes was 77 nm before loading the drug and the nanoparticles. By adding nanoparticles to the liposomes, the average size was increased to 131 nm. When DOX was loaded into the Lip-MNPs, the average size of the Lip-DOX-MNPs was increased to 460 nm with a uniform size distribution. The average size of MNPs was 35 nm, it almost corresponds to the result of TEM analysis. The zeta potential results are shown in Table 2. According to Table 2, all groups have a negative charge with a potential of -45.3 mV for Lip, -28.9 mV for Lip-MNPs and -6.9 mV for Lip-MNPs-DOX respectively. The zeta potential of Lip-MNPs-DOX and Lip-MNPs changed in a positive direction compared with that of empty liposomes that can be useful for improving penetration function of particles in tumor cell.Morphological characteristics of liposomes were assessed by TEM (Figure 2B). The spherical liposomes were between 70-100 nm in size (Figure 2B) that is consistent with the particle size found by DLS technique. To verify that enough Fe_2_O_3_ was loaded into the liposome, we used the ICP-OES method (Table 2). The loading percentages of the Lip-MNPs-DOX and Lip-MNPs groups were 18.52 and 35.94 respectively. HPLC analysis was used to evaluate the DOX concentration in liposomes. In Table 2, the results of this analysis are shown. The encapsulating efficiency of DOX was about 78% in Lip-DOX and 65% in Lip-MNPs-DOX indicating that DOX was efficiently loaded into the Liposomes. Release pattern of the DOX from Lip-MNPs-DOX and Lip-DOX is shown in Figure 3. For this purpose, two kinds of acidic (pH = 5.5) and neutral (pH = 7.4) media were tested.^42^ In fact release studies were simulated in acidic media, to assess the release pattern of DOX in the tumor tissue. In the drug release curves of the DOX composed biphasic, a severe initial release was followed by a stable release. Lip-MNPs-DOX release curve is slower than Lip-DOX due to the amount of magnetic nano particles in this group. Release pattern of DOX is slower in pH = 7 compared to pH = 5 in acidic media. The release pattern from the Lip-MNPs-DOX and the Lip-DOX was about 5% at pH 5.5 until 8 hours and thereafter the release rate was slower for all groups.

**Table 2:** Characteristic of samples

**Sample**	**Average size (nm)**	**Zeta potential (mV)**	**Polydispersity index**	**Loading % MNPs**	**Fe (ppm)**	**Loading % DOX**
Lip-MNPs-DOX	460	-6.9	0.45	18.52	377	65
Lip-DOX	179	-6.9	-	-	-	78
Lip-MNPs	131	-28.9	0.5	35.94	709	-
Lip	77	-45.3	0.6	-	-	-
MNPs	35	-43.1	0.4	-	-	-

###  SAR tests

**Figure 4:**
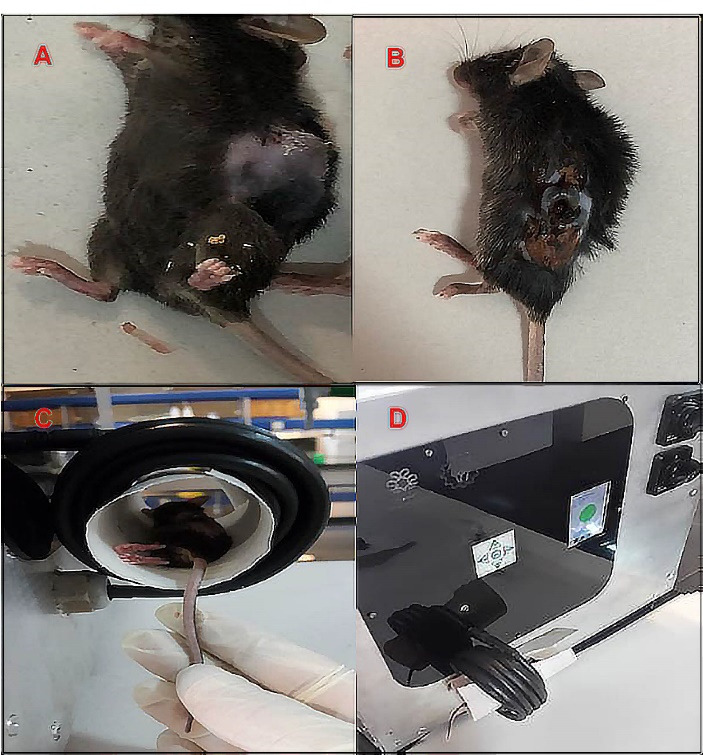
Tumor volume (A) before the treatment (B) 6 days after treatment with hyperthermia in Lip-MNPs-DOX group, (C, D) photograph of the hyperthermia device.

**Figure 5:**
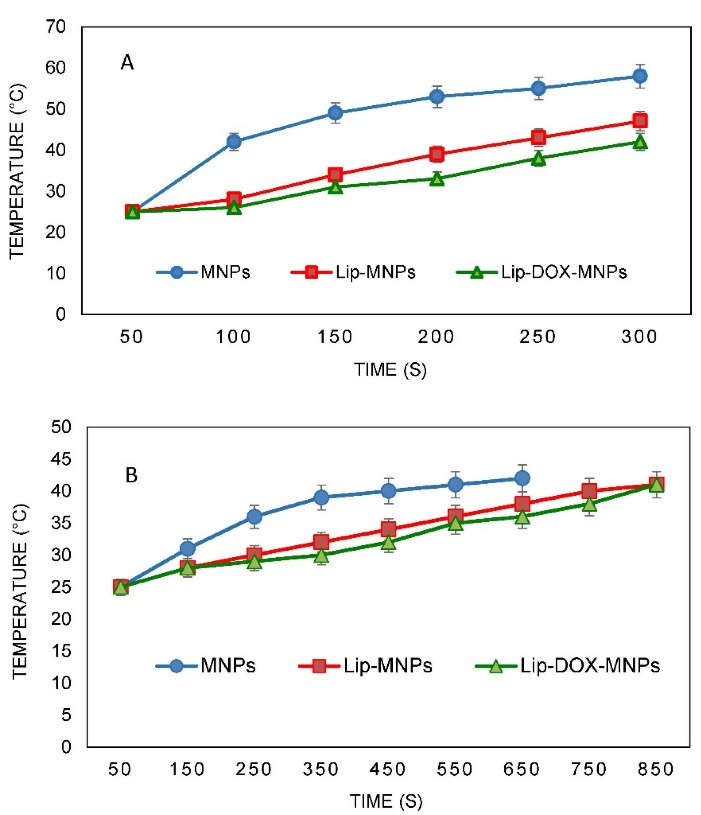
Magnetic heating curves for groups consist of magnetic nanoparticles (A) before in-vivo test under power of 1000 W and frequency of 405 kHz, (B) after injection in mice under power of 1000 W and frequency of 405 kHz. (The data represent mean ± standard deviation)

 Nanoparticle heat generation was measured in a hyperthermia device (Figure 4). The temperature rise as a function of time was measured by a thermometer inside the device coil. There was no temperature rise for groups without MNPs like Lip or DOX when they were exposed to an alternating magnetic field. Under the same device setting, the time required to reach a temperature of 42°C for Lip-DOX-MNPs, Lip-MNPs (0.5 cc of composition + 4 cc of citrate buffer) and MNPs (0.5 g of MNPs + 4 cc of citrate buffer) was recorded. For MNPs, there was rapid temperature rise from 25°C to 42°C in about 1 minute (Figure 5A). However the Lip-MNPs and Lip-MNPs-DOX needed longer times to reach 42°C possibly due to a lower dose of the nanoparticles in the two groups. The amount of heat produced by Lip-DOX-MNPs, Lip-MNPs was lower than that of the pure MNPs. These results are consistent with loading percentages MNPs in Table 2. To investigate the effect of trapping magnetic nano-particles by liposomes on heat generation, solutions of Lip-DOX-MNPs, Lip-MNPs and MNPs at the same concentration of MNPs (MNPs = 0.4 g + 4 cc of citrate buffer) were prepared. The temperature reached 42°C in 5 minutes in all groups. These results indicate that encapsulating MNPs in liposomes does not reduce heat generation. In other words, encapsulating Fe_3_O_4_ in liposomes showed good heat generation and confirmed Lip-MNPs-DOX to be effective for hyperthermia treatment. Finally, the injection was given to the mice and then they were exposed to a magnetic field for about 10-15 minutes to generate heat through a hyperthermia device. A temperature rise from 25°C to 42°C was observed in about 10 minutes in the groups containing MNPs (Figure 5B). To calculate the SAR, we obtained data from the temperature-time curves. The SAR depends on the concentration of the MNPs.

###  In vivo evaluation

**Figure 6:**
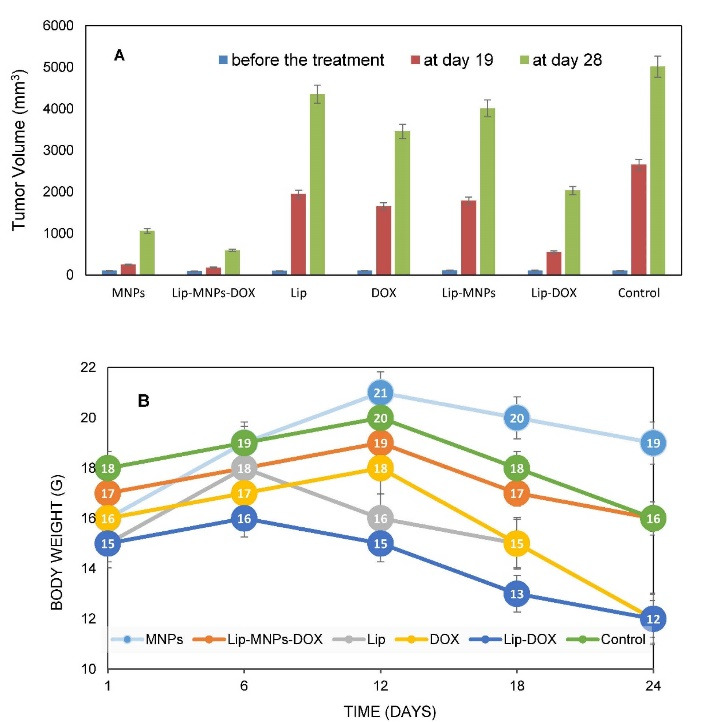
Tumor sizes in different groups at three conditions of measurement (n = 5 in each group and data was shown as mean ± standard deviation) (A), body weight after different treatments (B).

 For all groups, tumor size was measured and pathological tests were performed to evaluate the behavior of liposomes, nanoparticles, and the DOX in tumor treatment (Figure 6). Tumor sizes were measured every three days from the 13th day. The volume of tumors was calculated in different groups by measuring the length and width in the tumor. Before the treatment was started, tumor sizes were almost the same in all groups. According to numerical analysis in Figure 6A, the tumor volume in the control group increased from 980 mm^3^ on day 15 to 5000 mm^3^ on day 28 but in the Lip-MNPs-DOX group it increased less, from 200 mm^3^ on day 15 to 1000 mm^3^ on day 28. In the Lip-MNPs-DOX groups, the tumor growth was even slower than the other ones, reaching a maximum tumor volume below 1000 mm^3^. It seems that the presence of the chemotherapy drug and magnetic hyperthermia has prevented tumor growth (Figure 6A). The tumor volume remained less than 1000 mm^3^ in the MNPs groups with hyperthermia treatment compared to the other groups (Figure 6A). Superficial burns on the tumor were observed in this group. The tumor volume in the empty liposome group was about 5 times that in the other groups, indicating a non-therapeutic effect of the liposome in cancer similar to control groups without therapy. In the Lip-DOX groups, the tumor volume began to increase on day 20, which indicates that DOX inhibited tumor growth compared to the other groups, and thereafter showed a large increase in tumor volume, which may be prevented by injecting a higher dose of the drug (Figure 6A). In tumors of the Lip-MNPs group, the concentration of MNPs was less than in the MNPs group, and as a result the tumor volume was bigger. The antitumor efficacy of the Lip-MNPs was not significant, perhaps because the MNPs were stuck in the liposome and they could not come out of the liposome structure in 10 minutes. The tumor size of DOX groups was larger than the MNPs, Lip-MNPs and Lip-MNPs-DOX. However, the Lip-DOX and DOX groups showed maximum weight loss compared to the other groups that indicates the side effects of chemotherapy drugs against other cancer treatments (Figure 6B). There was no significant difference in body weights in the other groups. Generally, the tumor size increased in all groups when compared to measured before treatment. In the mice treated with hyperthermia, the tumors grew more slowly than in the DOX group. ON the 28th day, there were no deaths in any group but the long-term survival status of the Lip-MNPs-DOX, Lip-MNPs and Lip groups was better than that of the Lip-DOX and DOX groups. The results shown that the antitumor efficacy of the groups consisting of MNPs with hyperthermia was better than the other groups even the DOX groups.

**Figure 7:**
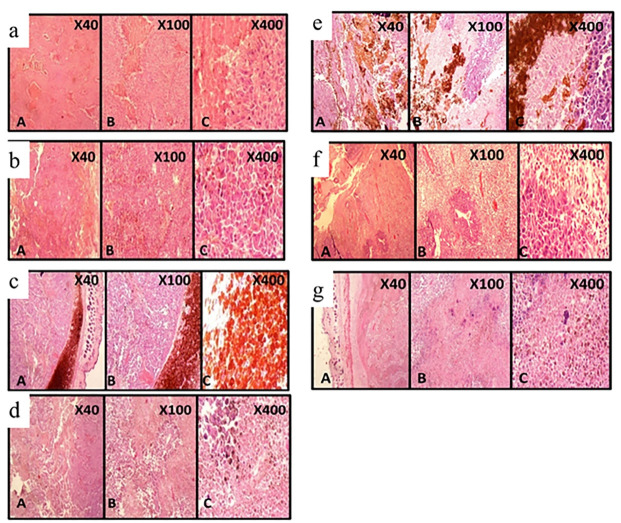
Histopathological of tumor section for different groups after 28 days: (a) Lip, (b) DOX, (c) MNPs, (d) Lip-MNPs, (e) Lip-DOX-MNPs, (f) Lip-DOX, (g) Control.

 The results of HE staining of tissue in different groups was studied (Figure 7). Next, the response to treatment was examined microscopically. In the Lip-MNPs-DOX groups, the maximum necrosis was 70% which being higher than other groups means that we have been able to prevent tumor growth. In the control and empty liposome groups mitosis was higher compared to treatment groups with hyperthermia and there was the lowest percentage of necrosis and the tissue structure of tumors was complete. For the groups with magnetite nanoparticles, the necrosis was more marked than in Lip-DOX, DOX, Lip and control groups. Also the microscopic investigation confirmed the presence of malignant melanoma in all groups.

 MNPs can convert their energy to heat under the influence of an alternating magnetic field.^13^ The previous studies have shown that heat-induced MNPs encapsulated in liposomes can be a good option for magnetic thermotherapy.^6,24,28^ The research in this field has investigated the effect of magnetic liposomes on the cancer treatment or drug loading on the liposome. Also, previous research has shown that with the encapsulation of DOX in liposomes the therapeutic efficacy did not dramatically increase. In the present study, we constructed novel compositions and evaluated Lip-MNPs-DOX antitumor efficacy by histological examination and the decrease in tumor growth after four rounds of administration to healthy mice. We combined these nanoparticles and DOX chemotherapy drug with biocompatible and biodegradable lipids that could enhance the localization of nanoparticles and DOX in the tumor region and this resulted in a higher percentage of necrosis and a decrease in tumor growth. In fact, our purpose in this research was to integrate two effective methods in cancer treatment and create a treatment with the least possible side effect. The tumor cells are more sensitive to heat than normal cells and this heat can destroy the tumor cells. Also heat generation assists the heat-sensitive liposomes and conversion of lipid layer to liquid phase and drug release. Because liposomes are lipid bilayer they can trap hydrophobic MNPs and DOX in their cores. Also, the heat generation by MNPs increased the permeability of tumor vessels, which facilitated drug delivery to the tumor tissue. We measured these MNPs ability to treat tumors in hyperthermia and drug delivery. First, a rotary evaporator was used to trap nanoparticles and to improve the loading percent of MNPs in liposomes an ultrasonic device was used. The results of Lip-MNPs-DOX analysis showed that an acceptable amount of DOX and nanoparticles were encapsulated in the liposome layers. Xenograft tumors in mice were induced by injection of B16/F10 cell line. To evaluate the effect of the chemotherapy drug and hyperthermia, we considered 7 different treatment groups. Then the tumor implantation was heated using hyperthermia to the power of 1000 Watts and a frequency of 405 kHz. The tumor volume in the mice receiving Lip-MNPs-DOX decreased severely compared to the other groups. The tumor volume at the end of the treatment in mice receiving Lip-MNPs-DOX was 9.29% that of the control and histological observation shown 70% necrosis. A noteworthy point was the lack of weight loss in the hyperthermia treatment groups, which is due to the use of low-dose chemotherapy drugs in this group (Figure 6B). The results confirmed that combining of DOX and MNPs in the tumor area will lead to effective cancer therapy.

## Conclusion

 Drug release in magnetic liposomes was improved with heat generated by nanoparticles. In vivo studies demonstrated the Lip-MNPs-DOX with suitable loading efficiencies are ideal carriers which could reduce the growth of malignant skin tumours and increase cancer cell necrosis without weight loss in treated groups of mice.

## Acknowledgments

 We appreciate the insights provided by Dr. Amir Reza Dehghanian about analyzing pathological tests, Mr. Omid Koohi about the in vivo section in Shiraz University of Medical Sciences, Ms. Khosravani for assistance in liposome synthesis, also Ms. Elisabeth Bamad Baker for manuscript edit in Exeter University of United Kingdom.

## Competing Interests

 The authors declare that they have no conflicts of interests. Authors disclose all relationships or interests that could have direct or potential influence or impart bias on the work.

## Ethical Approval

 Ethics approval and consent to participate our study was approved by the Iranian laboratory animal ethics framework under the supervision of the Iranian Society for the Prevention of Cruelty to Animals and Shiraz University Research Council (IACUC no: 4687/63). The recommendations of European Council Directive (2010/63/EU) of September 22, 2010, regarding the standards in the protection of animals used for experimental purposes, were also followed. The study was carried out in compliance with the ARRIVE guidelines.
